# Longitudinal survey of antibiotic stewardship practices in Wisconsin nursing homes, before and after a policy change

**DOI:** 10.1017/ash.2022.7

**Published:** 2022-03-14

**Authors:** Alissa E. Balke, Victoria L. Griffin, Diane Dohm, Brenda J. Ryther, Linda L. McKinley, Raymond P. Podzorski, Anna M. Marciniak, Christopher J. Crnich, Lindsay N. Taylor

**Affiliations:** 1 School of Medicine and Public Health, University of Wisconsin-Madison, Madison, Wisconsin; 2 Division of Quality Assurance, Wisconsin Department of Health Services, Milwaukee, Wisconsin; 3 Superior Health Quality Alliance, MetaStar, Madison, Wisconsin; 4 William S. Middleton Memorial VA Hospital, Madison, Wisconsin; 5 Microbiology Department, St Mary’s Hospital, Madison, Wisconsin; 6 Division of Public Health, Wisconsin Department of Health Services, Madison, Wisconsin; 7 Division of Infectious Disease, Department of Medicine, University of Wisconsin School of Medicine and Public Health, Madison, Wisconsin

## Abstract

The Centers for Medicare and Medicaid mandated that nursing homes implement antibiotic stewardship programs (ASPs) by November 2017. We conducted surveys of Wisconsin nursing-home stewardship practices before and after this mandate. Our comparison of these surveys shows an overall increase in ASP implementation efforts, but it also highlights areas for further improvement.

Antibiotic use in nursing homes (NHs) is high and frequently inappropriate.^1^ Antibiotic stewardship programs (ASPs), which aim to optimize antibiotic prescribing, are critical to combatting antibiotic resistance. The Centers for Medicare and Medicaid services (CMS) mandated that all NHs must implement an ASP by November 2017.^2^ This ASP requirement is based on the core elements of antibiotic stewardship for NHs developed by the Centers for Disease Control and Prevention (CDC).^3^ Recent assessments of ASPs in NHs have reported high levels of adherence to the CDC core elements^4,5^; however, knowledge of specific stewardship activities remains limited. In this study, we used survey data to detail the structure and process of ASPs in Wisconsin NHs before and after updates to CMS regulations.

## Methods

Between 2015 and 2018, 2 electronic surveys and 1 telephone survey were used to study Wisconsin NHs; they were completed by a facility representative (Supplementary Tables 1–3). Wisconsin Department of Health Services (DHS) survey 1 was electronically distributed through the Division of Quality Assurance listserv to all licensed NHs from February to May 2015 to capture information on antibiotic stewardship practices before the CMS mandate. Survey 2, a semistructured telephone survey designed to further characterize the structure and process of NH ASPs, was administered between January and April 2016 to a subset of respondents of Survey 1 who volunteered for further contact. Following the mandate, the Wisconsin DHS distributed survey 3, a structured electronic survey, to all licensed Wisconsin NHs from November to December 2018. Completion by the infection preventionist (IP) was recommended. Survey 3 posed the same questions as survey 1 and included elements from survey 2 to capture changes in the structure and processes of NH ASPs following new CMS regulations. Survey elements were encoded, and results were compiled to aid in analysis, which was performed in R version 1.3.1073 software (R Foundation for Statistical Computing, Vienna, Austria).

**Table 1.** Responding Nursing Home Characteristics

Variable	Survey Number and Year		
	Survey 1, 2015, No. (%)^a^	Survey 2, 2016, No. (%)^a^	Survey 3, 2018, No. (%)^a^
Respondents/Licensed nursing homes, no./total (%)	172/388 (44)	23/68 (34)	228/374 (60)^b^
Licensed beds, mean no. (SD)	110 (65)	144 (30)	79 (38)
**Facility ownership**	Count (%)	Count (%)	Count (%)
For profit	79 (46)	7 (30)	91 (40)
Nonprofit	69 (40)	9 (39)	112 (49)
Government	23 (13)	7 (30)	23 (10)
Veterans’ Affairs	1 (1)	0	2 (1)
**Facility affiliation**			
Independent	92 (53)	12 (52)	121 (53)
Multifacility organization	65 (38)	8 (35)	82 (36)
Hospital based	15 (9)	3 (13)	25 (11)
**Specialty resident services**			
Skilled nursing/short-term (subacute rehabilitation)	166 (97)	22 (96)	149 (98)
Dementia care	64 (37)	14 (61)	65 (43)
Intravenous care	122 (71)	21 (91)	132 (87)
Ventilator care	4 (2)	2 (9)	8 (5)
Tracheostomy care	80 (47)	14 (61)	78 (31)
Onsite wound care	142 (83)	21 (91)	133 (88)
Onsite phlebotomy	76 (44)	14 (61)	90 (59)
24-hour RN supervision	129 (75)	21 (91)	110 (72)

Note. SD, standard deviation; RN, registered nurse.

a Units unless otherwise specified.

b Compared to entire survey completion for surveys 1 and 2, 228 respondents completed a portion of survey 3 and 219 (34%) completed the entire survey.

## Results

The response rate to each survey is shown in Table 1. Profit status and affiliations were similar between NHs participating in surveys 1 and 3; NHs participating in survey 2 were larger with more resident services (Table 1).

### ASP structure

Prior to the release of new CMS regulations, 150 Wisconsin NHs (87%) reported engaging in stewardship activities; however, only 73 (49%) reported having a formally recognized ASP. Following the CMS mandate, all NHs participating in survey 3 reported having a formally recognized ASP.

Overall, director of nursing (N = 137, 91%), IP (N = 119, 79%), medical director (N = 114, 76%), and pharmacist (N = 112, 65%) were the roles most involved in stewardship efforts in 2015. Survey 2 revealed that the director of nursing and the IP were responsible for most stewardship activities. However, beyond attending ASP meetings, the medical director and the pharmacist had limited roles in conducting stewardship tasks.

**Fig. 1.**
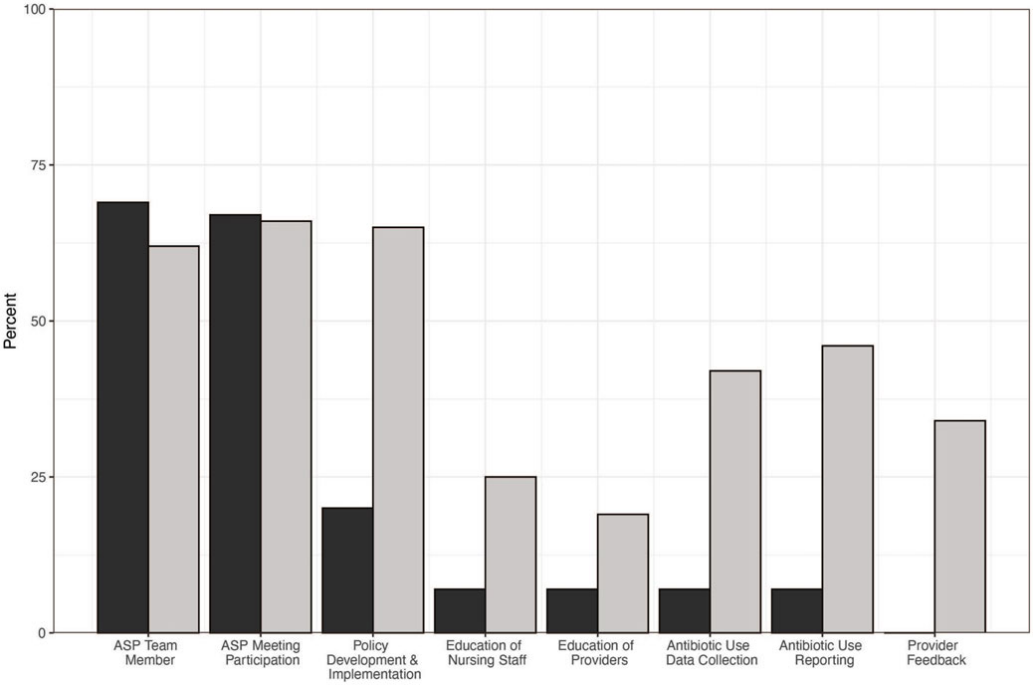
Pharmacist engagement in different antibiotic stewardship tasks before and after release of the revised Centers for Medicare and Medicaid regulations. Premandate involvement is displayed in black; postmandate involvement is displayed in grey.

Following the mandate, pharmacist (N = 79, 62%) and medical director (N = 100, 78%) involvement as members of the facility ASP team remained unchanged. Pharmacist involvement in stewardship tasks increased after the mandate (Fig. 1), with the largest increases in development of facility policies, tracking antibiotic use, and generating antibiotic-use reports. Although medical directors remained regular participants in ASP meetings (N = 70, 70%), their involvement in other critical antibiotic stewardship activities, including education of nursing staff (N = 7, 7%) and providers (N = 8, 8%) as well as participation in antibiotic utilization tracking and reporting (N = 9, 9%), remained quite low.

### ASP Process

The most-reported antibiotic-prescribing improvement strategy employed in Wisconsin NHs before the CMS regulations was review of antibiotic appropriateness performed at antibiotic start (N = 96, 65%) and/or following the return of culture results (N = 101, 89%). Some less commonly reported strategies included standardized antibiotic formularies (N = 11, 6%) and antibiotic preapproval (N = 11, 6%).

In survey 1, 126 NHs (73%) reported tracking antibiotic use, although 11 NHs (48%) in survey 2 reported the analysis of trends in these rates. For survey 1, 103 NHs (60%) reported tracking culture susceptibilities in some manner. In survey 2, only 9 NHs (39%) reported using a cumulative susceptibility report (ie, antibiogram). Tracking of other antibiotic use-related outcomes, such as antibiotic adverse events (N = 53, 31%) and *C. difficile* infections rates (N = 60, 35%), was less common.

Following the CMS mandate, antibiotic utilization tracking increased to 90% (N = 116). The proportion of NHs using rates increased to 63% (N = 72), and 66% (N = 76) reported trending antibiotic use over time. Tracking of antibiotic resistance using an antibiogram also increased (N = 27, 51.4%).

## Discussion

These surveys show increased implementation of stewardship activities in Wisconsin NHs following the release of the new CMS regulations. Notably, a high level of antibiotic stewardship activities existed in Wisconsin NHs prior to the CMS mandate, although only half had a formalized ASP. In a 2016 national survey of facilities participating in the CDC National Healthcare Safety Network, only 42% of participating NHs reported implementing all 7 CDC core elements of antibiotic stewardship.^5^ Other studies have reported significantly lower levels of stewardship activity among NHs prior to the new CMS regulations, although considerable geographic variation exists.^4^ The higher levels of self-reported stewardship activity identified in Wisconsin NHs in this study prior to the revised CMS regulations may reflect differences in survey design but may also be due to Wisconsin’s enhanced focus on medication safety education and survey activity since 2012. Similar to our findings, national levels of NH-reported antibiotic stewardship activity increased following release of the revised CMS regulations.^6^

Prior studies have identified significant relationships between the comprehensiveness of NH-reported antibiotic stewardship activity and IP certification and turnover.^4^ Here, we built upon these findings by showing that nursing staff, particularly the director of nursing and IP, were primarily responsible for the conduct of specific antibiotic stewardship tasks in Wisconsin NHs. Importantly, pharmacist engagement in stewardship activities increased significantly following the release of the revised CMS regulations. However, the revised CMS regulations appear to have had a minimal effect on the engagement of medical directors in stewardship activities, which remained disappointingly low.

Most NHs participating in national surveys report tracking antibiotic utilization and antibiotic use–related outcomes in some manner.^4–6^ Our findings are consistent with these national trends but suggest areas for continued improvement. Specifically, many NHs continue to track antibiotic utilization using count rather than rate metrics and do not identify trends in utilization data over time. Moreover, the current study shows that antibiogram use in NHs is increasing; however, much about antibiogram structure and its roles in stewardship operations and empiric antibiotic decision making remains unknown.

This study had several limitations. First, responses were self-reported and potentially affected by normative influences. Second, participation was voluntary with low response rate of licensed NHs, and participating NHs may differ from nonparticipating NHs. Taken together, our results may paint a relatively optimistic view of antibiotic stewardship activity in Wisconsin NHs. Although questions were coded, some differences between survey questions and modalities across time may limit comparability. Additionally, the survey instruments employed in the current study differed from those employed in other studies.^4–6^ Consequently, comparisons and contrasts drawn with these studies should be viewed with a certain level of caution.

## Supplementary material

For supplementary material accompanying this paper visit https://doi.org/10.1017/ash.2022.7. click here to view supplementary material

## References

[1] McElligott M , Welham G , Pop-Vicas A , Taylor L , Crnich CJ. Antibiotic stewardship in nursing facilities. Infect Dis Clin North Am 2017;31:619–638. 29079152 10.1016/j.idc.2017.07.008

[2] Centers for Medicare & Medicaid Services (CMS), HHS. Reform of requirements for long-term care facilities. Final Rule Fed Regist 2016;81:68688–68872. 27731960

[3] Core elements of antibiotic stewardship for nursing homes. Centers for Disease Control and Prevention website. https://www.cdc.gov/longtermcare/prevention/antibiotic-stewardship.html. Published June 11, 2020. Accessed December 17, 2020.

[4] Fu CJ , Mantell E , Stone PW , Agarwal M. Characteristics of nursing homes with comprehensive antibiotic stewardship programs, results of a national survey. Am J Infect Control. 2020;48:13–18. 31447117 10.1016/j.ajic.2019.07.015 PMC6935405

[5] Palms DL , Kabbani S , Bell JM , Anttila A , Hicks LA , Stone ND. Implementation of the core elements of antibiotic stewardship in nursing homes enrolled in the national healthcare safety network. Clin Infect Dis 2019;69:1235–1238. 30945729 10.1093/cid/ciz102

[6] Gouin KA , Kabbani S , Anttila A , et al. Implementation of core elements of antibiotic stewardship in nursing homes-National Healthcare Safety Network, 2016–2018. *Infect Control Hosp Epidemiol* 2021. doi: 10.1017/ice.2021.209. 34036926

