# Supplementary material for: Longitudinal survey of antibiotic stewardship practices in Wisconsin nursing homes, before and after a policy change
Source: Antimicrob Steward Healthc Epidemiol. 2022 Mar 14;2(1):e42. doi: 10.1017/ash.2022.7 (PMC9614914; doi:10.1017/ash.2022.7)
Supplement: Supplementary file 1 [file S2732494X22000079sup001.docx]

**Supplemental Materials**

**Table 1. Survey 1, 2015 Electronic Survey Tool**

| **Question Number** | **Question** | **Response** |
| --- | --- | --- |
| 1 | What state is your facility located in? (choose one) | (1) Nebraska  (2) Wisconsin |
| 2 | What zip code is your Long-Term Care Facility located in? | Free response |
| 3 | Please indicated the population size of the community where your facility is located: | (1) <10,000  (2) 10,000-50,000  (3) 50,001-200,000  (4) >200,000 |
| 4 | What is your primary position at the facility? | (1) Infection Control professional  (2) Staff Nurse  (3) Administrator  (4) Medical Director  (5) Pharmacist  (6) Other (free response) |
| 5 | How would you classify your facility? | (1) Skilled Nursing Facility  (2) Rehabilitation Facility |
| 6 | Facility affiliation: | (1) Independent (free-standing)  (2) Independent (within a retirement community)  (3) Multi-facility organization  (4) Hospital system (attached or within)  (5) Hospital system (free-standing) |
| 7 | What is the number of licensed beds at your facility? | Free response (integer) |
| 8 | What is your estimated daily census of residents? | Free response (integer) |
| 9 | Please indicate how many physicians (MD/DO) are responsible for antibiotic prescribing at your facility (Note: Only count those individuals who provide care for 3 or more residents): | Free response (integer) |
| 10 | Please indicate how many advance practitioners (NP/PA) are responsible for antibiotic prescribing at your facility (Note: Only count those individuals who provide care for 3 or more residents): | Free response (integer) |
| 11 | Does your facility have an antibiotic monitoring/stewardship program (ASP) or “antibiotic improvement team” (a person or group of persons involved in monitoring antibiotic use AND implementing programs to ensure appropriate use)? | (1) Yes  (2) No |
| 12 | Is this a formal (facility-recognized) or informal team? | (1) Formal  (2) Informal |
| 13 | Is this team a part of quality improvement or infection control efforts? | (1) Quality improvement  (2) Infection Control  (3) Both  (4) Neither  (5) Other (free response) |
| 14 | Which of the following individuals are involved in your facility’s antimicrobial stewardship/improvement efforts? (Please select all that apply? | (1) Infection Control Professional  (2) Director of Nursing  (3) Medical Director  (4) Pharmacist  (5) Administrator  (6) Other (free response) |
| 15 | How is pharmacy support provided at your facility? | (1) In house  (2) Independent consultant  (3) Large national consulting firm (i.e. Omnicare)  (4) We do not have pharmacy support  (5) Other (free response) |
| 16 | Is the pharmacist who supports your facility involved in your antibiotic stewardship/improvement efforts? | (1) Yes  (2) No |
| 17 | Has there been training and education regarding appropriate antibiotic use at your facility within the last 12 months? | (1) Yes  (2) No |
| 18 | To whom was this training directed? | (1) Physicians  (2) Physician assistants  (3) Pharmacists  (4) Nurses  (5) Certified Nursing Assistants  (6) Other (free response) |
| 19 | Please indicate all formats the training was provided in: | (1) Live lectures  (2) Printed materials  (3) Online courses  (4) Other (free response) |
| 20 | Do families and/or residents receive any information or education on appropriate antibiotic use? | (1) Yes (2) No |
| 21 | Does your facility employ a systematic process to track antibiotic use of all or specific antibiotics over time (ad hoc review of an antibiotic during inter-professional rounds does not qualify)? | (1) Yes  (2) No |
| 22 | What measures of antibiotic use are utilized in your facility? | (1) New antibiotic starts  (2) Antibiotic duration  (3) Antibiotic days of therapy (DOT)  (4) Antibiotic defined daily dose (DDD)  (5) Antibiotic costs  (6) Use of specific antibiotic classes (e.g. fluoroquinolones) or agents (e.g. ciprofloxacin)  (7) Amount of antibiotic prescribed by individual providers (e.g. Dr. Smith ordered 5 antibiotic prescriptions in May, 3 in June, etc.)  (8) Other (free response) |
| 23 | How are lab services directed at your facility? | (1) Onsite Lab  (2) Contracted offsite lab  (3) Other (free response) |
| 24 | Are urinary dipstick tests permitted in your facility? | (1) Yes  (2) No |
| 25 | Please select the answer that best describes your facility’s policies and procedures regarding urinary dipstick testing in the evaluation of a resident with a change in condition: | (1) There are no formal policies or procedures  (2) Existing policies and procedures encourage urinary dipstick testing  (3) Existing policies and procedures discourage urinary dipstick testing but this process is not actively observed/measured  (4) Existing policies and procedures discourage urinary dipstick testing and this process is actively observed/measured |
| 26 | What type of antibiogram (summary table of antibiotic sensitivities from bacteria at your facility) is used in your facility? | (1) Facility generated antibiogram  (2) Reference laboratory generated antibiogram  (3) We do not have an antibiogram  (4) Other (free response) |
| 27 | Does your facility have an antibiotic formulary (only certain agents are available for use)? | (1) Yes  (2) No |
| 28 | Who decides what agents will be included in the formulary? | (1) facility-based committee  (2) Parent organization or facility chain  (3) Affiliated hospital  (4) Accountable Care Organizations  (5) Other (free response) |
| 29 | Does your facility employ any local guidelines, treatment algorithms, or antibiotic use policies to help guide providers treatment of commonly encountered infections? | (1) Yes  (2) No |
| 30 | What commonly encountered infections are included in these local guidelines/algorithms or policies? | (1) Vancomycin resistant enterococci (VRE)  (2) Methicillin resistant Staphylococcus aureus (MRSA)  (3) Clostridium difficile  (4) Multi-drug resistant gram-negative bacilli (e.g. Acinetobacter and Pseudomonas)  (5) Asymptomatic bacteriuria/Urinary Tract Infections  (6) Pneumonia  (7) Skin and soft tissue infections  (8) Other (free response) |
| 31 | What topics do these local guidelines/algorithms or policies address? | (1) Prioritizes antibiotics for common infections (e.g. nitrofurantoin is first line for cystitis)  (2) Defines length of therapy for common infections  (3) Defines appropriate testing for common infections  (4) Defines signs/symptoms of common infections (Loeb or McGeer)  (5) Defines when it is appropriate to employ watchful waiting  (6) Other (free response) |
| 32 | After a resident is admitted to your facility, does your facility require “preapproval” of certain antibiotics (i.e. preapproval means the antibiotic cannot be prescribed until someone give specific permission)  ? | (1) Yes  (2) No |
| 33 | Please list antibiotics that require preapproval: | Free response |
| 34 | Reviewing the appropriateness of an antibiotic after is has been prescribed (post-prescribing antibiotic review) is a strategy that some nursing homes have employed to reduce inappropriate antibiotic use. Please select the activity level that best describes the extent of post-prescribing review in your facility: | (1) Level 1: post-prescribing review of antibiotic appropriateness is performed inconsistently or not at all.  (2) Level 2: appropriateness of antibiotics is assessed consistently during inter-disciplinary resident care rounds but facility staff do not routinely engage providers when inappropriate use is identified.  (3) Level 3: appropriateness of antibiotics is assessed regularly during inter-disciplinary resident care rounds and facility staff consistently engage providers when inappropriate use is identified. |
| 35 | When antibiotics are monitored to determine if they are appropriate, at which point is that evaluated? | (1) start of therapy  (2) at some specific time (for example at 3 or 7 days)  (3) After culture results return  (4) Other (free response) |
| 36 | When antibiotics are monitored to determine if they are appropriate, who determines antibiotic appropriateness at your facility? | (1) Medical director  (2) Pharmacist  (3) Outside Infectious Disease Consultant  (4) Other (free response) |
| 37 | Please indicate whether use of each specific antibiotic is monitored or reviewed for appropriate use: | (1) Quinolones  (2) Linezolid  (3) Oral Cephalosporins  (4) Trimethoprim/sulfamethoxazole  (5) Nitrofurantoin  (6) Other (free response) |
| 38 | In the last 24 months, has your facility targeted any specific pathogen, antibiotic, or practices for improvement? Please specify yes or no and then comment on what was done |  |
| 38a | Asymptomatic bacteriuria | (1) Yes (free response)  (2) No |
| 38b | C. difficile infection | (1) Yes (free response)  (2) No |
| 38c | Pneumonia | (1) Yes (free response)  (2) No |
| 38d | Skin and soft tissue infection | (1) Yes (free response)  (2) No |
| 38e | Other | (1) Yes (free response)  (2) No |
| 39 | Does your antimicrobial stewardship program or institution monitor any outcomes related to antimicrobial use? | (1) Antibiotic adverse events  (2) Resistant pathogen isolation rates (VRE, MRSA, Gram-negative)  (3) C. difficile rates  (4) Antibiotic resistance  (5) Compliance with guidelines  (6) Other (free response)  (7) We do not currently track any of these outcomes |
| 40 | If you have instituted an antibiotic stewardship program, have you measured improvement in any of the following? | (1) Specified antibiotic prescriptions  (2) Antibiotic days of therapy  (3) Antibiotic duration  (4) Antibiotic starts  (5) Antibiotic costs  (6) Antibiotic adverse events  (7) Inappropriate antibiotic use  (8) Resistant pathogen isolation rates (VRE, MRSA, Gram negative)  (9) C. difficile rates  (10) Antibiotic resistance  (11) Other (free response)  (12) We do not currently track any of these outcomes  (13) Do not have an antibiotic stewardship program |
| 41 | How big of a problem is inappropriate use in your facility (please select the number that best reflects your assessment from the scale below)? | Slider labels: Not a problem, Is a moderate problem, is our biggest problem |
| 42 | If you do not already have an antibiotic monitoring/stewardship program do you plan to initiate one within the next year? | (1) Yes  (2) No  (3) Already have one |
| 43 | What, if any, barriers prevent improvements in antibiotic use in your facility? | (1) Lack of funding/resources  (2) Administration has not made it a priority at my facility  (3) Our medical director has not made it a priority  (4) Practitioners are resistant to input on prescribing practices  (5) Lack of personnel to develop a program  (6) Pharmacist has not made it a priority at my facility  (7) Limited familiarity or access to computers of quality improvement tools (e.g., unable to use spreadsheets to collect an trend process and outcome data)  (8) Our reference laboratory is unwilling to provide aggregated reports on facility microbiology data (antibiogram)  (9) Lack of access to an infectious disease expert  (10) Other (free response) |
| 44 | Would you allow the investigators of this survey study to contact you via telephone to get more detail on how antibiotic use is tracked and strategies employed to improve antibiotic use in your facility? | (1) Yes (free response of contact information)  (2) No |

**Table 2. Survey 2, 2016 Follow up Telephone Interview Tool**

*Respondent facility demographic information linked from 2015 survey responses.*

| **Question number** | **Question Text/Script** | **Response** |
| --- | --- | --- |
| Record ID from Survey 1 linked to these responses | | |
| 1 | To start I am going to ask you about your facility's antimicrobial stewardship or antibiotic use improvement team: Does your facility have an Antimicrobial Stewardship Program or "antibiotic use improvement team"? | (1) Yes  (2) No |
| 2 | Who of the following individuals participate in the antibiotic use improvement team? (check all that apply) | (1) Infection Control Professional  (2) On-site Pharmacist  (3) Consultant Pharmacist  (4) Medical Director  (5) Other Physician  (6) Other LTCF Administrator  (7) Director of Nursing  (8) Other |
| 3 | In which of the following antibiotic stewardship activities is the Infection Control Professional involved? (check all that apply) | (1) Active participation in quality improvement meetings in which antibiotic stewardship are discussed  (2) Development and implementation of policies on antibiotic prescribing practices  (3) Provides structured education to nursing staff about antibiotic prescribing (4) Provides structured education to providers about antibiotic prescribing  (5) Data collection of antibiotic utilization patterns and/or antibiotic resistance rates  (6) Data analysis of antibiotic utilization patterns and/or antibiotic resistance rates  (7) Audit and feedback to nursing staff about antibiotic use patterns  (8) Audit and feedback to providers about antibiotic use patterns  (9) Other (free response) |
| 4 | In which of the following antibiotic stewardship activities is the on-site pharmacist involved? (check all that apply) | (1) Active participation in quality improvement meetings in which antibiotic stewardship are discussed  (2) Development and implementation of policies on antibiotic prescribing practices  (3) Provides structured education to nursing staff about antibiotic prescribing (4) Provides structured education to providers about antibiotic prescribing  (5) Data collection of antibiotic utilization patterns and/or antibiotic resistance rates  (6) Data analysis of antibiotic utilization patterns and/or antibiotic resistance rates  (7) Audit and feedback to nursing staff about antibiotic use patterns  (8) Audit and feedback to providers about antibiotic use patterns  (9) Other (free response) |
| 5 | In which of the following antibiotic stewardship activities is the consultant pharmacist involved? (check all that apply) | (1) Active participation in quality improvement meetings in which antibiotic stewardship are discussed  (2) Development and implementation of policies on antibiotic prescribing practices  (3) Provides structured education to nursing staff about antibiotic prescribing (4) Provides structured education to providers about antibiotic prescribing  (5) Data collection of antibiotic utilization patterns and/or antibiotic resistance rates  (6) Data analysis of antibiotic utilization patterns and/or antibiotic resistance rates  (7) Audit and feedback to nursing staff about antibiotic use patterns  (8) Audit and feedback to providers about antibiotic use patterns  (9) Other (free response) |
| 6 | In which of the following antibiotic stewardship activities is the medical director involved? (check all that apply) | (1) Active participation in quality improvement meetings in which antibiotic stewardship are discussed  (2) Development and implementation of policies on antibiotic prescribing practices  (3) Provides structured education to 7nursing staff about antibiotic prescribing (4) Provides structured education to providers about antibiotic prescribing  (5) Data collection of antibiotic utilization patterns and/or antibiotic resistance rates  (6) Data analysis of antibiotic utilization patterns and/or antibiotic resistance rates  (7) Audit and feedback to nursing staff about antibiotic use patterns  (8) Audit and feedback to providers about antibiotic use patterns  (9) Other (free response) |
| 7 | In which of the following antibiotic stewardship activities is another physician involved? (check all that apply) | (1) Active participation in quality improvement meetings in which antibiotic stewardship are discussed  (2) Development and implementation of policies on antibiotic prescribing practices  (3) Provides structured education to nursing staff about antibiotic prescribing (4) Provides structured education to providers about antibiotic prescribing  (5) Data collection of antibiotic utilization patterns and/or antibiotic resistance rates  (6) Data analysis of antibiotic utilization patterns and/or antibiotic resistance rates  (7) Audit and feedback to nursing staff about antibiotic use patterns  (8) Audit and feedback to providers about antibiotic use patterns  (9) Other (free response) |
| 8 | In which of the following antibiotic stewardship activities is the LTCF administrator involved? (check all that apply) | (1) Active participation in quality improvement meetings in which antibiotic stewardship are discussed  (2) Development and implementation of policies on antibiotic prescribing practices  (3) Provides structured education to nursing staff about antibiotic prescribing (4) Provides structured education to providers about antibiotic prescribing  (5) Data collection of antibiotic utilization patterns and/or antibiotic resistance rates  (6) Data analysis of antibiotic utilization patterns and/or antibiotic resistance rates  (7) Audit and feedback to nursing staff about antibiotic use patterns  (8) Audit and feedback to providers about antibiotic use patterns  (9) Other (free response) |
| 9 | In which of the following antibiotic stewardship activities is the Director of Nursing involved? (check all that apply) | (1) Active participation in quality improvement meetings in which antibiotic stewardship are discussed  (2) Development and implementation of policies on antibiotic prescribing practices  (3) Provides structured education to nursing staff about antibiotic prescribing (4) Provides structured education to providers about antibiotic prescribing  (5) Data collection of antibiotic utilization patterns and/or antibiotic resistance rates  (6) Data analysis of antibiotic utilization patterns and/or antibiotic resistance rates  (7) Audit and feedback to nursing staff about antibiotic use patterns  (8) Audit and feedback to providers about antibiotic use patterns  (9) Other (free response) |
| 10 | In which of the following antibiotic stewardship activities is this “other” individual involved? (check all that apply) | (1) Active participation in quality improvement meetings in which antibiotic stewardship are discussed  (2) Development and implementation of policies on antibiotic prescribing practices  (3) Provides structured education to nursing staff about antibiotic prescribing (4) Provides structured education to providers about antibiotic prescribing  (5) Data collection of antibiotic utilization patterns and/or antibiotic resistance rates  (6) Data analysis of antibiotic utilization patterns and/or antibiotic resistance rates  (7) Audit and feedback to nursing staff about antibiotic use patterns  (8) Audit and feedback to providers about antibiotic use patterns  (9) Other (free response) |
| 11 | Next I am going to ask about your Medical Director:  How long has your current medical director been in his/her position? | (1) < 1 year  (2) 1-2 years  (3) 3-4 years  (4) > 5 years |
| 12 | Is your medical director a Certified Medical Director (CMD; certification obtained through the American Medical Directors Association)? | (1) Yes  (2) No |
| 13 | Next, I am going to ask you about your facility's pharmacist and their role in antibiotic use improvement efforts. First, I am interested in knowing if you have a facility-employed pharmacist, or if you have a contracted pharmacist? Does your facility have an on-site pharmacist? | (1) Yes  (2) No |
| 14 | Does your facility have pharmacy technicians? | (1) Yes  (2) No |
| 15 | If yes, however many individuals are involved in pharmacy services? | Numeric free response |
| 16 | In some facilities, pharmacists perform advanced antibiotic review. This may look like reviewing indications for antibiotic prescriptions, culture data and susceptibilities. Based this description, does your pharmacist perform advanced antibiotic review? | (1) Yes  (2) No |
| 17 | Please identify which of the following is representative of pharmacist's advanced review of antibiotics at your facility: (check all that apply) | (1) Review of antibiotic indication  (2) Review of culture susceptibilities to make sure the proper antibiotic is being administered  (3) Review of duration of therapy to minimize excessive length of therapy  (4) Flagging of antibiotic prescriptions that do not have an indication  (5) Other (free response) |
| 18 | How often is this advanced review practice performed? | (1) With every antibiotic prescribed  (2) Intermittently |
| 19 | If advanced review of antibiotics reveals an error of prescribing, how is the prescriber notified? | Free response |
| 20 | I'm interested in learning about how antibiotic use is tracked at your facility. Tracking of antibiotic use means collecting and aggregating the data in a report to be looked at over time. This is different from noting that a resident is on antibiotics on a given day. Based on this description, is antibiotic use tracked at your facility by a standard method? | (1) Yes (free response)  (2) No |
| 21 | Antibiotic use measures can be presented as raw data or as an adjusted rate. Raw data is generated by adding up the total number of antibiotic starts observed in a pre-specified time period (for example, 7 antibiotic starts in the month of December). An adjusted rate is generated by dividing the number of antibiotic starts observed in a pre-specified time period by the total number of resident days in that same time period (For example, 5.5 antibiotic starts per 1,000 resident days in the month of December).  Is the tracked data of antibiotic use presented in raw format or adjusted for resident census? | (1) Raw data  (2) Standardized data |
| 22 | How frequently is antibiotic use data reviewed? | (1) monthly  (2) quarterly  (3) biannually  (4) annually  (5) never |
| 23 | By whom is the antibiotic use data reviewed? | (1) Infection Control Professional  (2) Medical Director  (3) Antibiotic use improvement team or QAPI committee  (4) Nursing Director  (5) Providers  (6) Our facility does not review these data |
| 24 | I am next interested in learning about how your facility tracks antibiotic-related outcomes like antibiotic-resistant infections. Are antibiotic-related outcomes tracked at your facility (prompt with an example if the subject is unsure)? | (1) Yes  (2) No |
| 25 | Please specify which of the following outcomes are tracked in your facility (please select all that apply): | (1) Clostridium difficile infection  (2) MRSA infection  (3) Resistant gram negative bacteria (e.g. ESBL or Fluoroquinolone resistance)  (4) Other types of resistant bacteria (free text)  (5) Adverse drug events  (6) Other outcomes (free text) |
| 26 | Clinical outcomes can be presented as raw data or as an adjusted rate. As a reminder, raw data would simply be the total number of a specific event observed in a per-specified time period (for example, 9 cases of Clostridium difficile in the month of December). If these data were converted to an adjusted rate then the total number of events in a pre-specified time period would be divided by the number of resident days in that same time period (for example, 6.5 Clostridium difficile infections per 1,000 resident days).  Is the tracked data of antibiotic use presented in raw format or adjusted for resident census? | (1) Raw format  (2) Adjusted for resident census |
| 27 | How frequently is clinical outcome data reviewed? | (1) monthly  (2) quarterly  (3) biannually  (4) annually  (5) never |
| 28 | By whom is the clinical outcome data reviewed? | (1) Infection Control Professional  (2) Medical Director  (3) Antibiotic use improvement team or QAPI committee  (4) Nursing Director  (5) Providers  (6) Our facility does not review these data |
| 29 | An antibiogram is a profile of the level of resistance to different types of antibiotics among the bacteria recovered from cultures in your facility. An example of an antibiogram would be the proportion of E. coli that was resistant to ciprofloxacin over the last 6 months. An antibiogram is NOT the same as the susceptibility report from an individual culture.  Is an antibiogram used in your facility? | (1) Yes  (2) No |
| 30 | How is the antibiogram created? | (1) From culture data specific to only this facility  (2) From pooled culture data from multiple facilities using the same reference laboratory  (3) Unsure |
| 31 | How often is the antibiogram updated? | (1) Monthly  (2) Every 6 months  (3) Yearly  (4) Every 2 years  (5) Every 5 years  (6) Unsure |
| 32 | Please describe how the antibiogram is used: | Free text |
| 33 | How is the antibiogram made available? (check all that apply) | (1) Electronically  (2) Paper |
| 34 | Do you think providers in your facility are aware of antibiogram results? | (1) Yes  (2) No  (3) Unsure |
| 35 | Do you think the antibiogram information has an impact on the antibiotic prescribing choices of providers in your facility? | (1) Yes  (2) No  (3) Unsure |
| 36 | Are education or educational materials on appropriate antibiotic use provided? | (1) Yes  (2) No |
| 37 | To whom is education on appropriate antibiotic use provided? | (1) Prescribers  (2) Nursing staff  (3) Residents/Families  (4) Other (free text) |
| 38 | How is education made available to prescribers? | (1) Verbal informal lessons  (2) Lectures  (3) Newsletters  (4) Posters  (5) Online modules  (6) Other (free text) |
| 39 | How is education made available to nursing staff? | (1) Verbal informal lessons  (2) Lectures  (3) Newsletters  (4) Posters  (5) Online modules  (6) Other (free text) |
| 40 | How is education made available to residents/families? | (1) Verbal informal lessons  (2) Lectures  (3) Newsletters  (4) Posters  (5) Online modules  (6) Other (free text) |
| 41 | How is education made available to other? | (1) Verbal informal lessons  (2) Lectures  (3) Newsletters  (4) Posters  (5) Online modules  (6) Other (free text) |
| 42 | What content has been addressed in education? (check all that apply) | (1) Risk of antibiotic side effects  (2) Risk of future infection with antibiotic resistant with inappropriate antibiotic use  (3) Risk of C. difficile with inappropriate antibiotic use  (4) Statement that watchful waiting (not prescribing antibiotics and clinically monitoring for new or worsening symptoms) is not the same as "doing nothing"  (5) Other (free text) |
| 43 | Does your facility employ antibiotic use guidelines, algorithms, or policies to help guide providers' antibiotic prescribing practices? | (1) Yes  (2) Other |
| 44 | What aspects of antibiotic prescribing are addressed by the guidelines, algorithms or policies in your facility? | (1) Treatment of specific infections (e.g., UTI)  (2) Avoiding use of certain antibiotics (e.g., Fluoroquinolones)  (3) Duration of antibiotics  (4) Other (free text) |
| 45 | How were these antibiotic use guidelines, algorithms, or policies developed? | (1) Created locally  (2) Provided by parent organization  (3) Other (free text) |
| 46 | How are these antibiotic use guidelines, algorithms, or policies made available to providers? | (1) Pocket cards  (2) Pamphlets and/or handouts  (3) Electronically  (4) Incorporated into antibiotic order forms  (5) Other (free text) |
| 47 | Does your facility employ standardized processes to communicate a change in a resident's condition to providers? | (1) Yes  (2) No |
| 48 | Is this a standardized tool or form? (such as INTERACT) | (1) Yes (free text)  (2) No |
| 49 | Is the use of this standardized communication tool tracked? | (1) Yes (free text)  (2) No |
| 50 | Does someone, other than the prescribing provider, review antibiotic orders to determine if they are being prescribed appropriately? | (1) Yes  (2) No |
| 51 | What antibiotic orders are reviewed for appropriateness? | (1) All antibiotic orders  (2) Some antibiotic orders (free text description) |
| 52 | Who is responsible for reviewing the appropriateness of antibiotic orders in your facility (select all that apply)? | (1) Nurse caring for the resident  (2) Nurse manager or nurse supervisor  (3) Infection control nurse  (4) Director of nursing  (5) Pharmacist  (6) Medical director  (7) Other (free text) |
| 53 | What criteria are used to determine if an antibiotic order is appropriate? | (1) Loeb criteria  (2) Original McGeer criteria (1991)  (3) Modified McGeer criteria (2012)  (4) Other (free text) |
| 54 | If an antibiotic order is deemed not appropriate, how is it dealt with in your facility? | Free text |
| 55 | Are the overall numbers and/or rate of inappropriate antibiotic orders measured and reviewed by facility leadership on a regular basis? | (1) Yes  (2) No |
| 56 | Please share anything else about the review of antibiotic appropriateness in your facility we have not yet covered: | Free text |
| 57 | The antibiotic timeout is a process in which residents who are currently receiving an antibiotic are reassessed to determine if their existing antibiotic orders need to be changed, typically 48 to 72 hours after starting treatment. FOR THE PURPOSE OF THIS QUESTION, changing an antibiotic because resistance was identified on culture results IS NOT CONSIDERED PART OF AN ANTIBIOTIC TIMEOUT. Antibiotic timeouts are only considered to have occurred when the review is used to switch residents to narrow-spectrum antibiotics, shorten duration of treatment or to stop antibiotics altogether.  Are antibiotic timeouts routinely used in your facility as an antibiotic stewardship strategy in your facility? | (1) Yes  (2) No |
| 58 | Please estimate how often antibiotic timeouts are performed in your facility: | (1) < 25% of antibiotic orders  (2) 26-50% of antibiotic orders  (3) 51 - 75% of antibiotic orders  (4) >75% of antibiotic orders |
| 59 | Please describe which individuals are typically involved in the antibiotic timeout when performed at your facility: | Free text |
| 60 | Please select what is the focus of the typical antibiotic timeout at your facility (select all that apply): | (1) Stopping the antibiotic  (2) Changing to an antibiotic with more narrow spectrum  (3) Shortening duration of treatment  (4) Other (free text) |
| 61 | Please describe any tools or instruments used to help guide antibiotic timeout discussions: | Free text |
| 62 | Does your facility provide prescribers (physicians, nurse practitioners, and physician assistants) with reports of their individual prescribing patterns and/or use of diagnostic tests? | (1) Yes  (2) No |
| 63 | What do these reports focus on? | (1) frequency of antibiotic prescribing  (2) frequency of prescribing certain antibiotics or antibiotic classes  (3) frequency of inappropriate antibiotic prescribing  (4) frequency of ordering urine cultures  (5) other (free text) |
| 64 | Do these reports include comparisons to other prescribers in the facility? | (1) Yes  (2) No |
| 65 | Who prepares these reports? | Free text |
| 66 | How often are these reports generated and provided to the prescriber? | Free text |

**Table 3. Survey 3, 2018 Electronic Survey Tool**

| **Question Number** | **Question** | **Response** |
| --- | --- | --- |
| Facility Demographics | | |
| 1 | Facility Ownership: | (1) For profit  (2) Not for profit  (3) Government  (4) Veteran’s Affairs |
| 2 | Facility Affiliation: | (1) Independent, free standing  (2) Independent, within a continuing care retirement community  (3) Multi-facility organization (chain/corporation)  (4) Hospital system, attached  (5) Hospital system, free-standing |
| 3 | Total number of beds: | Free text, numeric |
| 4 | Average annual percent occupancy | Free text, numeric |
| 5 | Which of the following resident services are delivered in your facility? | (1) Long-term custodial care  (2) Skilled nursing/short-term (subacute rehabilitation  (3) Care for residents with dementia in specialized unit or designated cluster of beds  (4) IV infusion using central lines  (5) Management of residents on a ventilator  (6) Management of residents with a tracheostomy  (7) Dedicated staff to provide wound care  (8) Dedicated staff to perform blood draws  (9) 24-hour a day on-site supervision by an RN  (10) Other (free text) |
| Infection Control FTE | | |
| 6 | How many full-time employees (FTEs) are currently dedicated to your facility’s IC program? | Free text, numeric |
| 7 | What is the highest level of professional training of the individual primarily responsible for the infection control program in your facility? | (1) CNA  (2) LPN  (3) RN  (4) MD  (5) No FTEs are dedicated to infection control  (6) Other, please specify |
| 8 | From question #7, how many years has he/she been in that position at your facility? | Free text, numeric |
| 9 | From question #7, how many years of experience does he/she have doing IC-related work? | Free text, numeric |
| 10 | From question #7, has he/she received any specific IC training? | (1) Certified in Infection Control (CIC)  (2) State or local training course with certificate  (3) No specific infection control training  (4) Other (free text) |
| 11 | From question #7, is coordination of ICC this individual’s full time or part time role? | (1) Full time  (2) Part time |
| 12. | If part-time, please indicate which of the activities listed below he/she also performs: | (1) Facility administration (i.e. Director of Nursing (DON))  (2) Quality manager  (3) Staff education/staff development  (4) Employee health  (5) Direct resident care  (6) Other (free text) |
| 13 | On average, during a normal work week (40 hours), what percent of time is spent performing all infection control related activities? | Free text, numeric |
| 14 | Given the total time spent on infection control-related activities, please indicate the approximate percentage of time (in an average 40 hour work week) spent on these specific activities (sum to 100%) | (1) infection surveillance  (2) infection control policy development  (3) Staff education  (4) monitoring adherence to policy  (5) Employee health activities  (6) Other (free text) |
| IC Committee | | |
| 15 | Is there a committee in your facility that reviews IC related activities (such as reports, policies and procedures, etc.)? If the answer is no, go to #19. | (1) Yes  (2) No |
| 16 | If YES, how frequently does this committee meet? | (1) Annually  (2) Quarterly  (3) Monthly  (4) Weekly  (5) Other (free text) |
| 17 | If YES, indicated the members represented on the committee (check all that apply) | (1) Facility board members  (2) Nursing administrators (i.e., DON, ADON)  (3) Medical Director  (4) Quality department  (5) Pharmacy Department  (6) Environmental services  (7) Unit managers or supervisors  (8) Physician staff  (9) Nursing staff  (10) Other (free text) |
| Infection control activities | | |
| 18 | Our facility uses standard definitions (such as McGeer criteria or CDC NHSN definitions) to determine if a resident has an infection. | (1) Yes  (2) No |
| 19 | Our facility uses new antibiotic prescription (starts) to determine if a resident has an infection. | (1) Yes  (2) No |
| 20 | Our facility reviews provider notes to determine if a resident has an infection | (1) Yes  (2) No |
| 21 | Our facility maintains a list of residents with healthcare associated infection in a log book. | (1) Yes  (2) No |
| 22 | Our facility keeps a record of healthcare associated infections in an electronic spreadsheet or database | (1) Yes  (2) No |
| 23 | Our facility performs house-wide surveillance of infections among our residents | (1) Yes  (2) No |
| 24 | Our facility performs targeted surveillance for specific infections among our residents | (1) Yes  (2) No |
| 25 | Our facility tracks rates of infection over time to identify trends (e.g., monthly rate, quarterly rate, annual rate). | (1) Yes  (2) No |
| 26 | Our facility creates summary reports (e.g., trends) of healthcare associated infections. | (1) Yes  (2) No |
| 27 | Our facility reports rates of specific infections (e.g., # UTIs/1000 resident days/month) | (1) Yes  (2) No |
| 28 | Our facility shares infection surveillance data with facility board members | (1) Yes  (2) No |
| 29 | Our facility shares infection surveillance data with facility leadership (i.e., CEO, COO, DON, ADON, Medical Director). | (1) Yes  (2) No |
| 30 | Our facility shares infection surveillance data with all facility nursing staff. | (1) Yes  (2) No |
| 31 | Our facility shares infection surveillance data with all physicians providing care to residents. | (1) Yes  (2) No |
| 32 | Our facility measures adherence to hand hygiene policies in at least one patient care are by staff observation. | (1) Yes  (2) No |
| 33 | Our facility measures adherence to isolation precautions among staff (i.e., the percentage of those who comply with wearing of gloves or donning of gowns). | (1) Yes  (2) No |
| 34 | Our facility infection control personnel monitor/observe environmental cleaning practices to ensure consistent cleaning and disinfection practices are followed. | (1) Yes  (2) No |
| 35 | Our facility has a specific person (or people) responsible for reviewing antibiotic utilization | (1) Yes  (2) No |
| 36 | Our facility restricts the use of specific antibiotics | (1) Yes  (2) No |
| 37 | Our facility shares adherence rates to specific policies (e.g., hand hygiene) with all staff. | (1) Yes  (2) No |
| 38 | The infection control program coordinator is responsible for employee health policies | (1) Yes  (2) No |
| 39 | Employee immunizations are tracked by the infection control program. | (1) Yes  (2) No |
| 40 | Our facility requires staff to have immunization or proof of immunity to hepatitis B. | (1) Yes  (2) No |
| 41 | Our facility requires staff to have immunization or proof of immunity to varicella (chickenpox). | (1) Yes  (2) No |
| 42 | Our facility requires staff to have immunization or proof of immunity for measles/mumps/rubella (MMR). | (1) Yes  (2) No |
| 43 | Our facility requires staff to receive vaccination for seasonal influenza. | (1) Yes  (2) No |
| 44 | Our facility provides staff with seasonal influenza vaccine at no cost to them. | (1) Yes  (2) No |
| 45 | Our facility requires staff to be screen for tuberculosis (e.g., PPD skin test) at time of employment. | (1) Yes  (2) No |
| 46 | Facility staff receive training and education on infection control issues. | (1) Strongly agree  (2) Agree  (3) Neutral  (4) Disagree  (5) Strongly disagree |
| 47 | Facility staff have knowledge about infection control issues. | (1) Strongly agree  (2) Agree  (3) Neutral  (4) Disagree  (5) Strongly disagree |
| 48 | Infection control is a priority in our facility. | (1) Strongly agree  (2) Agree  (3) Neutral  (4) Disagree  (5) Strongly disagree |
| 49 | Our facility has resources to support its infection control program. | (1) Strongly agree  (2) Agree  (3) Neutral  (4) Disagree  (5) Strongly disagree |
| 50 | Preventing healthcare associated infections among residents in our facility is a challenge. | (1) Strongly agree  (2) Agree  (3) Neutral  (4) Disagree  (5) Strongly disagree |
| 51 | Staff turnover has an impact on the prevention of healthcare-associated infections in our facility. | (1) Strongly agree  (2) Agree  (3) Neutral  (4) Disagree  (5) Strongly disagree |
| 52 | Which of the following healthcare-associated infections is the **greatest challenge** for your facility at this time (please select only one)? | (1) Clostridium difficile associated diarrhea  (2) Catheter-associated urinary tract infections  (3) Central-line associated blood stream infections  (4) Methicillin-resistant S. aureus infections  (5) Multidrug-resistant gram negative bacteria  (6) Vancomycin-resistant Enterococcus  (7) Norovirus  (8) Influenza  (9) Other (free text) |
| 53 | Which aspect of infection control is most challenging for your facility at this time (please select only one)? | (1) Environmental cleaning  (2) Hand hygiene  (3) Infection surveillance (tracking or tending resident infections)  (4) Isolation precautions/managing resident with MDROs  (5) Laundry/linen handling  (6) Outbreak management  (7) Other (free text) |
| 54 | What external sources of information are used by your facility to address infection-control related questions (please check all that apply)? | (1) American Medical Directors Association (AMDA) resources  (2) Association for Professionals in Infection Control and Epidemiology (APIC) resources  (3) Centers for disease control and prevention (CDC) guidelines/website  (4) Corporate organization resources (if applicable)  (5) Health Department  (6) Hospital infection control personnel (local but not affiliated with your facility)  (7) Infection control consultant (external contract)  (8) National Associated of Directors of Nursing Administration (NADONA) resources  (9) Other (free text) |
| 55 | Provider you level of interest in the following areas:  Improving hand hygiene in the long-term care facility | (5) Very relevant  (4) Relevant  (3) Neutral  (2) Irrelevant  (1) Very irrelevant |
| 56 | Practical approaches to infectious disease surveillance in long-term care facilities | (5) Very relevant  (4) Relevant  (3) Neutral  (2) Irrelevant  (1) Very irrelevant |
| 57 | Outbreak identification and management in long-term care facilities | (5) Very relevant  (4) Relevant  (3) Neutral  (2) Irrelevant  (1) Very irrelevant |
| 58 | Building an infection control program in long-term care facilities | (5) Very relevant  (4) Relevant  (3) Neutral  (2) Irrelevant  (1) Very irrelevant |
| 59 | Management of multi-drug resistant organisms (MDROs) in long-term care facilities | (5) Very relevant  (4) Relevant  (3) Neutral  (2) Irrelevant  (1) Very irrelevant |
| 60 | Vaccine preventable illnesses among long-term care residents | (5) Very relevant  (4) Relevant  (3) Neutral  (2) Irrelevant  (1) Very irrelevant |
| 61 | Prevention of CAUTI in long-term care facilities | (5) Very relevant  (4) Relevant  (3) Neutral  (2) Irrelevant  (1) Very irrelevant |
| 62 | Preventing respiratory tract infection (RTIs) in long-term care facilities | (5) Very relevant  (4) Relevant  (3) Neutral  (2) Irrelevant  (1) Very irrelevant |
| 63 | Environmental cleaning in long-term care facilities | (5) Very relevant  (4) Relevant  (3) Neutral  (2) Irrelevant  (1) Very irrelevant |
| 64 | Infection control issues in assisted living facilities | (5) Very relevant  (4) Relevant  (3) Neutral  (2) Irrelevant  (1) Very irrelevant |
| 65 | Antibiotic stewardship in long-term care facilities | (5) Very relevant  (4) Relevant  (3) Neutral  (2) Irrelevant  (1) Very irrelevant |
| 66 | Practical quality improvement in long-term care facilities | (5) Very relevant  (4) Relevant  (3) Neutral  (2) Irrelevant  (1) Very irrelevant |
| Antibiotic Stewardship | | |
| 67 | Is the pharmacist who supports your facility a member of your antibiotic stewardship team? | (1) Yes  (2) No |
| 68 | Which of the following antibiotic stewardship activities is the pharmacist involved in? (Check all that apply) | a. Participates in facility antibiotics stewardship meetings  b. Participates in review and development of antibiotic use and stewardship policies  c. Provides in-service training to facility nursing staff that focuses on antibiotic prescribing issues  d. Provides in-service training to providers focusing on antibiotic prescribing issues  e. Provides family education that focuses on antibiotic-related issues and policies  f. Is involved in collection of data on facility antibiotic utilization patterns and/or antibiotic resistance rates  g. Is involved in generating reported on facility antibiotic utilization patterns and/or antibiotic resistance rates.  h. Is involved in antibiotic reviews to determine if existing antibiotic orders are appropriate (only select this option if the pharmacist is reviewing resident records and/or talking with providers to determine if there is a clinical justification for the existing antibiotic orders).  i. Is involved in antibiotic reviews to determine if existing antibiotic orders should be modified (only select this option if the pharmacist is reviewing the resident microbiology data and providing recommendation for modification of existing antibiotic orders)  j. Manages uncomplication infection within parameters of a formal facility physician/pharmacist collaborative practice agreement as allowed by Wisconsin State Statute.  k. Other |
| 69 | Is the medical director of your facility a member of your antibiotic stewardship team? | (1) Yes  (2) No |
| 70 | Which of the following antibiotic stewardship activities is the facility medical director involved in? (Check all that apply) | a. Participates in facility antibiotics stewardship meetings  b. Participates in review and development of antibiotic use and stewardship policies  c. Provides in-service training to facility nursing staff that focuses on antibiotic prescribing issues  d. Provides in-service training to providers focusing on antibiotic prescribing issues  e. Provides family education that focuses on antibiotic-related issues and policies  f. Is involved in generating reported on facility antibiotic utilization patterns and/or antibiotic resistance rates.  g. Provides as-needed feedback to individual providers when problematic antibiotic prescribing and other infection management patterns are identified.  h. Provides regular feedback to individual providers about their antibiotic prescribing and other infection management patterns (only select this option if there is a formal process for characterizing and delivering the feedback – report card).  i. Other |
| 71 | Is surveillance for antibiotic use currently performed in your facility? | (1) Yes  (2) No |
| 72 | [If yes to #71]  Which types of antibiotic use are tracked in your facility? | (1) all antibiotic orders are tracked  (2) Other |
| 73 | [If yes to #71]  Which antibiotic use measures are employed in your facility (more than one option may be selected): | (1) Antibiotic starts per specified time period (e.g., month, quarter, year)  (2) Antibiotic days of therapy per specified time period (e.g., month, quarter, year)  (3) Other – write in |
| 74 | In the facility antibiotic use reports, are antibiotic measures presented as a raw number (e.g., 8 antibiotic starts in the month of September) or as a rate (5.3 antibiotic starts per 1,000-resident days)? | (1) rate  (2) raw number |
| 75 | Are antibiotic use measures trended (i.e., are antibiotic use measure from previous time periods included in the same facility antibiotic use report) in your facility? | (1) yes, data on antibiotic use measures from previous time periods are included in a table format  (2) No  (3) Yes, data on antibiotic measures from previous time periods are included in a figure (e.g., bar chart) format |
| 76 | Are any target or benchmarks incorporated in your facility antibiotic use reports (e.g., the previous year’s average antibiotic starts per month was 7.5 and the current month is 9)? | (1) yes  (2) no |
| 77 | Does the facility antibiotic use report stratify antibiotic measures (e.g., by type of infection, by appropriateness)? | (1) yes  (2) no |
| 78 | Which types of characteristics are used to stratify the antibiotic measure in the facility report (more than one option may be selected)? | (1) By type of infection (e.g. urinary tract infections, pneumonia, etc.)  (2) By type of antibiotic (e.g., fluoroquinolones)  (3) By appropriateness (e.g., urinary tract infections meeting vs. not meeting McGeer criteria)  (4) By duration (e.g., treatment events 7 days)  (5) By location (e.g., Unit A vs. Unit B) |
| 79 | Using the description of surveillance provided at the beginning of this section, are any antibiotic-related outcomes tracked in your facility? | (1) yes  (2) no |
| 80 | Please select which antibiotic-related outcomes are tracked in your facility (select all that apply): | (1) Individual types of resistant organisms are tracked (e.g., methicillin-resistant Staphylococcus aureus (MRSA), Clostridium difficile, etc.)  (2) Antibiogram  (3) Types of resident side effects (e.g., antibiotic-associated diarrhea, skin reactions, etc.) |
